# Indoor mold odor in the workplace increases the risk of Asthma-COPD Overlap Syndrome: a population-based incident case–control study

**DOI:** 10.1186/s13601-019-0307-2

**Published:** 2020-01-15

**Authors:** Maritta S. Jaakkola, Taina K. Lajunen, Jouni J. K. Jaakkola

**Affiliations:** 1 Center for Environmental and Respiratory Health Research (CERH), University of Oulu, Faculty of Medicine, Aapistie 5B, P.O.Box 5000, 90014 Oulu, Finland; 2 Medical Research Center Oulu, Oulu University Hospital and University of Oulu, Oulu, Finland

**Keywords:** Asthma-COPD Overlap Syndrome, Case–control study, Exposure to mold odor, Occupational exposure, Population-based study

## Abstract

**Background:**

Previous studies have suggested an increased risk of asthma related to indoor dampness problems, but their role in the etiology of Asthma-COPD Overlap Syndrome has not been studied. We utilized a population-based incident case–control study to assess potential effect of indoor dampness and molds at home and at work on development of ACOS.

**Methods:**

We recruited systematically all new cases of asthma diagnosed during a 2.5-year study period (1997–2000) and randomly selected controls from the source population of adults 21–63 years old and representing 500,000 persons-years in the Pirkanmaa Hospital District, South Finland. Exposure indicators included water damage, damp stains or paint peeling, visible mold, and mold odor, asked separately for home and workplace. The clinically diagnosed case series consisted of 521 adults with newly diagnosed asthma. Altogether 25 of them satisfied the criteria for ACOS-cases, i.e. FEV1/FVC < 0.70 in post-bronchodilator spirometry. The control series, including 932 controls, were from a random sample of source population, after excluding 76 (7.5%) controls with asthma.

**Results:**

In logistic regression analysis adjusting for confounders, the risk of ACOS was significantly related to presence of mold odor in the workplace (OR 3.43; 95% CI 1.04–11.29), but not to other dampness indicators. The fraction of ACOS attributable to workplace mold odor was 70.8% (95% CI 3.8–91.1%) among the exposed. The risk of ACOS was not related to mold exposures at home.

**Conclusions:**

Present results provide new evidence of the significant relation between workplace exposure to mold odor and adult-onset ACOS.

## Background

The recent identification of existence of concomitant asthma and COPD, called the Asthma-COPD Overlap Syndrome (i.e. ACOS) among adult obstructive lung diseases [1] raises the question, what determines development of this disease when compared to development of asthma only. We observed in our population-based study of adult-onset asthma in Southern Finland that ACOS was quite common among adults with newly diagnosed asthma [2]. In this Finnish Environment and Asthma Study (FEAS) we found that current smoking increased the risk of ACOS 7.9-fold and former smoking 3.2-fold compared to non-smokers. Thus, prevention of smoking is an important measure to prevent development of the irreversible obstruction characterizing ACOS. In addition, we identified in our recent literature search (up to January 2019) a study from Canada that reported exposure to air pollution, namely PM2.5 and ozone, to be a risk factor for ACOS [3]. Another recent study among World Trade Center-exposed firefighters in September 11, 2001, provided evidence that a massive irritant exposure may increase the risk of asthma and COPD as well as ACOS [4, 5].

We were interested if there are other environmental determinants for ACOS, which could be subject to preventive actions. We have previously reported from FEAS that the presence of visible mold and/or mold odor in the workplace increases significantly the risk of incident asthma, with an adjusted odds ratio of 1.54 (95% CI 1.01–2.32) [6]. In addition, exposure to mold odor was related to reduced lung function levels among those with recently diagnosed asthma [7], the effect on FEV1 being on average − 240 ml (95% CI − 0.48 to − 0.003). These findings formed the background for this investigation assessing indoor dampness and mold problems as determinants of ACOS.

## Methods

### Study design

We conducted a large population-based incident case–control study on risk factors for adult-onset asthma in Southern Finland (i.e. the Finnish Environment and Asthma Study, FEAS) in 1997–2000 [6]. We systematically recruited all the new cases of asthma and randomly selected controls among working-aged adults in a geographically defined area, Pirkanmaa Hospital District (population 440,913 in 1997). In the present study we focused on a specific subgroup of cases, i.e. on those who had also COPD when asthma was diagnosed, i.e. ACOS cases [2]. The criterion for COPD was based on an acceptable post-bronchodilator spirometry showing FEV1/FVC less than 70% of predicted. A total of 390 (74.9%) of the original 521 cases had sufficient spirometry testing conducted to assess potential existence of ACOS.

### Cases

To be identified as an ACOS case, the subject had to have newly-diagnosed asthma with the spirometry showing obstruction and significant reversibility with bronchodilator medication [6]. In addition, the post-bronchodilator spirometry had to show persistent obstruction with FEV1/FVC less than 0.70 [2]. Thus, among the 390 working-aged adults with newly-diagnosed asthma and post-bronchodilator spirometry, there were 25 cases of ACOS, giving the prevalence of 6.4% (95% CI 4.9–8.8%).

### Controls

The controls were the original 932 population-based controls drawn from the source population that produced the cases of asthma, including both ACOS cases and asthma-only cases [6].

### Ethics approval

The FEAS study had been approved in 1997 by the Ethics Committee of the Finnish Institute of Occupational Health (where the Principal Investigator Dr MS Jaakkola was employed at the time of the data collection of FEAS) and the Ethics Committee of the Pirkanmaa Hospital District (approval number 97172 Jaakkola M). All participants gave an informed consent.

### Exposure assessment

The exposures of interest were occurrence of indoor dampness and mold problems at home or at work or both, based on questionnaire information given just before the time when the study subjects underwent the diagnostic tests for asthma or ACOS.

We inquired about exposures during the past 12 months and/or in the past 1–3 years and/or over 3 years ago, except that for mold odor we included only occurrence during the past 12 months. For mold odor we gave the following options: almost daily, 1–3 days a week, 1–3 days a month, < 1 day a month, or never. In the present analyses, we applied any time as the time frame for the occurrence of indoor dampness and mold problems, and compared it to never.

### Statistical analyses

To study potential effects of indoor dampness and mold problems on occurrence of ACOS and asthma-only, we applied multinomial logistic regression by SAS procedure LOGISTIC with glogit link function, using SAS statistical package v.9.4 (SAS Institute Inc., Cary, NC, USA). We adjusted these relations for gender, age, education and smoking as core covariates. When we adjusted the analyses further for parental atopy, keeping pets, and occupational exposures other than mold exposure, the point estimates of ACOS did not change more than 10%. Thus, we decided to present the results adjusted for the core covariates.

## Results

### Characteristics of the study population

The study population for this study on determinants of ACOS included a total of 390 subjects with adult-onset asthma and acceptable post-bronchodilator spirometry. Among them 365 (93.6%) had asthma only, while 25 (6.4%, 95% CI 4.9–8.8%) had ACOS. Table 1 shows comparison of these two groups with the controls. ACOS cases were more frequently men, and they were older and had lower education compared to both asthma-only cases and controls. ACOS-cases were clearly more often current smokers, somewhat more often former smokers, and more commonly exposed to second-hand smoke. In contrast, asthma-only cases had more pet exposure at any point in time.

**Table 1:** Characteristics of Asthma-only and ACOS cases and population-based controls, the Finnish Environment and Asthma Study

Characteristic	Asthma-only (N = 365)	ACOS (N = 25)	Controls (N = 932)
Gender			
Men, no. (%)	120 (32.9)^a^	17 (68.0)	438 (47.0)
Women, no. (%)	245 (67.1)^a^	8 (32.0)^b^	494 (53.0)
Age, years			
20–29, no. (%)	87 (23.8)	0 (0.0)	141 (15.1)
30–39, no. (%)	80 (21.9)	2 (8.0)	224 (24.0)
40–49, no. (%)	85 (23.3)	6 (24.0)	254 (27.3)
50–59, no. (%)	88 (24.1)	10 (40.0)	240 (25.8)
60–63, no. (%)	25 (6.9)	7 (28.0)	73 (7.8)
Education			
Other, no. (%)	72 (19.7)	10 (40.0)	158 (17.0)
Vocational course, no. (%)	57 (15.6)	8 (32.0)	104 (11.2)
Vocational institution, no. (%)	109 (29.9)	1 (4.0)	271 (29.1)
College-level education, no. (%)	82 (22.5)	3 (12.0)	261 (28.0)
University or corresponding, no. (%)	45 (12.3)	3 (12.0)	138 (14.8)
Parental asthma or allergy^c,d^, no. (%)	128 (35.1)	9 (36.0)	204 (21.9)
Smoking^e^			
Never, no. (%)	180 (49.3)	2 (8.0)	485 (52.0)
Former, no. (%)	83 (22.7)	7 (28.0)	205 (22.0)
Current, no. (%)	100 (27.4)	16 (64.0)	240 (25.8)
Second-hand smoke during past 12 months, at home or at work, no. (%)	75 (20.6)	8 (32.0)	166 (20.6)
Having pets at some point, no. (%)	262 (71.8)	15 (60.0)	616 (66.1)

Definition of abbreviations: ACOS, Asthma-COPD Overlap Syndrome; NA, Not applicable

^a^A GWAS analysis revealed that one woman with asthma-only had accidentally been recorded as a man during the data entry process, her gender record was corrected for this analysis

^b^One woman with ACOS was in a GWAS analysis found to be of Asian ethnicity and was excluded due to lack of appropriate reference population for lung function measures

^c^Parental asthma missing for 33 asthma-only cases, 3 ACOS cases and 90 controls

^d^Parental allergy missing for 33 asthma-only cases, 3 ACOS cases and 90 controls

^e^Smoking information missing for 2 asthma-only cases and 2 controls

### Exposure indicators

Table 2 presents exposure to indoor dampness and molds among the ACOS cases, asthma-only cases and controls. ACOS cases were less often exposed to any water damage, any dampness, and any visible molds than asthma-only cases or controls. In contrast, ACOS cases experienced more exposure to mold odor at work (16.0%) compared to asthma-only cases (11.2%) and controls (9.9%).

**Table 2:** Distribution of exposure indicators in asthma-only and ACOS cases and controls, the Finnish Environment and Asthma Study

Exposure indicator	Location	Asthma only (N = 365)	ACOS (N = 25)	Controls (N = 932)
Any water damage	At home, no. (%)	41 (11.4)	0 (0.0)	103 (11.2)
	At work, no. (%)	45 (12.3)	1 (4.0)	121 (13.0)
	At home and/or at work, no. (%)	74 (20.5)	1 (4.2)	206 (22.4)
Any dampness	At home, no. (%)	73 (20.0)	3 (12.0)	185 (19.9)
	At work, no. (%)	64 (17.5)	2 (8.0)	171 (18.4)
	At home and/or at work	119 (32.6)	5 (20.0)	310 (33.4)
Any visible mold	At home, no. (%)	17 (4.7)	0 (0.0)	56 (6.0)
	At work, no. (%)	23 (6.3)	0 (0.0)	42 (4.5)
	At home and/or at work, no. (%)	39 (10.7)	0 (0.0)	94 (10.1)
Any mold odor	At home, no. (%)	36 (9.9)	2 (8.0)	86 (9.2)
	At work, no. (%)	41 (11.2)	4 (16.0)	86 (9.2)
	At home and/or at work, no. (%)	66 (18.1)	6 (24.0)	156 (16.7)
Any visible mold or mold odor	At home, no. (%)	46 (12.6)	2 (8.0)	118 (12.7)
	At work, no. (%)	48 (13.2)	4 (16.0)	99 (10.7)
	At home and/or at work, no. (%)	82 (22.5)	6 (24.0)	193 (20.7)

*ACOS* Asthma-COPD Overlap Syndrome

### Relations between dampness and mold exposures and ACOS

Table 3 shows results from the multinomial logistic regression analyses investigating potential effects of different types of dampness and mold exposures on the risk of ACOS compared to the controls, adjusting for the core covariates gender, age, education, and smoking. The risk of ACOS was significantly increased in relation to exposure to mold odor at work, with an OR of 3.43 (95% CI 1.04–11.29). Adjustment for additional covariates, including parental atopy, SHS, pets, and occupational exposures, changed the OR less than 10%, OR being 3.39. The fraction of ACOS attributable to workplace mold odor was 70.8% (95% CI 3.8–91.1%) among the exposed. Combined exposure to visible mold and/or mold odor at work was also related to an increased risk of ACOS, with the adjusted OR of ACOS 3.17 (95% CI 0.97–10.37). The point estimates for risks of asthma-only were around or below 1, apart from adjusted OR related to visible mold being 1.33 (95% CI 0.78–2.27).

**Table 3:** The Relation between exposure indicators of indoor dampness and mold problems and the risk of ACOS and asthma only, the Finnish Environment and Asthma Study

Exposure indicator	Location	Outcome	Unadjusted	Adjusted for gender, age, education, and smoking
			OR (95% CI)	OR (95% CI)
Any water damage	At home	Asthma only	1.02 (0.69–1.49)	1.04 (0.70–1.54)
		ACOS	NA	NA
	At work	Asthma only	0.94 (0.65–1.36)	0.91 (0.63–1.32)
		ACOS	0.28 (0.04–2.08)	0.51 (0.07–4.05)
	At home and/or at work	Asthma only	0.89 (0.66–1.21)	0.89 (0.65–1.20)
		ACOS	0.15 (0.02–1.12)	0.23 (0.03–1.76)
Any dampness	At home	Asthma only	1.01 (0.74–1.36)	1.01 (0.74–1.37)
		ACOS	0.55 (0.16–1.85)	0.60 (0.17–2.08)
	At work	Asthma only	0.95 (0.69–1.30)	0.91 (0.66–1.26)
		ACOS	0.39 (0.09–1.66)	0.70 (0.16–3.13)
	At home and/or at work	Asthma only	0.97 (0.75–1.25)	0.95 (0.73–1.23)
		ACOS	0.50 (0.19–1.34)	0.68 (0.25–1.90)
Any visible mold	At home	Asthma only	0.77 (0.44–1.34)	0.75 (0.43 1.32)
		ACOS	NA	NA
	At work	Asthma only	1.43 (0.84–2.41)	1.33 (0.78–2.27)
		ACOS	NA	NA
	At home and/or at work	Asthma only	1.07 (0.72–1.59)	1.02 (0.68–1.53)
		ACOS	NA	NA
Any mold odor	At home	Asthma only	1.08 (0.72–1.62)	1.03 (0.68–1.57)
		ACOS	0.86 (0.20–3.69)	0.98 (0.21–4.52)
	At work	Asthma only	1.25 (0.84–1.85)	1.15 (0.77–1.72)
		ACOS	1.87 (0.63–5.58)	3.43 (1.04–11.29)^a^
	At home and/or at work	Asthma only	1.10 (0.80–1.51)	1.03 (0.74–1.42)
		ACOS	1.57 (0.62–4.00)	2.37 (0.86–6.54)
Any visible mold or mold odor	At home	Asthma only	0.99 (0.69–1.43)	0.96 (0.66–1.40)
		ACOS	0.60 (0.14–2.57)	0.75 (0.17–3.38)
	At work	Asthma only	1.27 (0.88–1.84)	1.19 (0.82–1.73)
		ACOS	1.60 (0.54–4.76)	3.17 (0.97–10.37)
	At home and/or at work	Asthma only	1.11 (0.83–1.48)	1.06 (0.78–1.42)
		ACOS	1.21 (0.48–3.07)	1.95 (0.72–5.30)

*ACOS* Asthma-COPD Overlap Syndrome, *CI* confidence interval, *NA* not applicable, *OR* odds ratio

^a^Statistically significant (p < 0.05)

### Potential joint effect of mold odor with smoking and parental atopy

We explored potential interaction, i.e. joint effects of mold odor and smoking, because both were identified as independent determinants for ACOS. These analyses had smaller numbers of those with both exposures. There was clear indication that the effect of mold odor was stronger among smokers compared to non-smokers (Table 4). Further, there was some indication that the effect of mold odor was stronger among subjects with no parental atopy.

**Table 4:** Exposure to mold odor and the risk of ACOS according to potential effect modifiers, the Finnish Environment and Asthma Study

Potential effect modifier	Unadjusted OR (95% CI)	Adjusted OR (95% CI)
Parental atopy		
Yes	0.94 (0.11–7.83)	1.29 (0.15–11.42)^a^
No	3.12 (0.83–11.66)	3.64 (0.95–14.03)^a^
Smoking		
Never	< 1.00^b^	< 1.00^b^
Current	3.03 (0.79–11.67)	5.68 (1.22–26.41)^c^

*ACOS* Asthma-COPD Overlap Syndrome, *CI* confidence interval, *OR* odds ratio

^a^Adjusted for age and gender

^b^No. of cases exposed to mold odor = 0

^c^Adjusted for age, gender and education

## Discussion

We conducted a large population-based incident case–control study, the Finnish Environment and Asthma Study, in Southern Finland in 1997–2000 [6]. The study size corresponds to a follow-up of approximately 100,000 individuals for 5 years, if we assume a realistic asthma incidence of 1 case per 1000 person-years. We found that 6.4% of those with newly-diagnosed adult-onset asthma had actually ACOS, which is a disease entity that was discovered after the data collection of the original FEAS study had been conducted. We have previously reported that both active smoking and ex-smoking are significant risk factors for ACOS [2], and in this study we investigated if indoor dampness or mold problems also form a risk factor for ACOS. The prevalence of ACOS was in this study slightly smaller than that we reported previously [2], because we have now performed a genome-wide analysis for the data, and detected that one woman with ACOS was of Asian ethnicity. She was excluded because she would need a different set of reference values for spirometry.

We found that exposure to mold odor at work increased significantly the risk of ACOS, the OR being 3.43 after adjusting for the core covariates. Further adjustment for additional potential confounders did not influence the point estimate more than 10%, the OR being 3.39. The fraction of ACOS attributable to workplace mold odor was as high as 70.8% among the exposed.

We found that the effect of mold odor on ACOS was stronger among current smokers, suggesting a synergistic effect between these exposures. Similarly, the risk increase in relation to mold odor was present mainly among those with no history of parental atopy. Thus, we hypothesize that those with atopic genetic propensity would develop asthmatic inflammation when exposed to indoor molds, while those with no such genetic predisposition would react to such exposure with different type of inflammation leading to persistent obstruction.

### Validity of results

In the original large population-based FEAS, we were able to recruit a high proportion of the newly-diagnosed cases of asthma (86%) in the Pirkanmaa study area in Southern Finland, through a thorough recruitment in the whole health care system (response rate 90%) and with the help of the National Social Insurance Institution (NSII) (response rate 78%). The health insurance provided by the NSII warranted special (i.e. higher) reimbursement of asthma medications for those whose asthma was diagnosed according to the national Finnish standards, which warranted a special incentive for study subjects to have their asthma diagnosed. NSII invited those participants who had newly-diagnosed asthma in the study area, but who had not yet participated in our study.

The response rate among the controls was also reasonably high: 67% of the total control sample and 80% of those who had a phone number in the Pirkanmaa area, so among those who were likely to actually live in their Pirkanmaa address during the study period. Thus, any major selection bias in participation in our study is unlikely.

To reduce potential misclassification of mold exposures which were assessed based on questionnaire-reporting, we introduced this study to the participants as a study on environmental factors and asthma in general, with no special focus on mold or dampness problems. Several studies have compared occupant-reporting of the presence of indoor dampness and mold problems with findings in building inspections or measurements of fungi in indoor dust, as presented in the review by Jaakkola and Jaakkola [8]. Most of them have demonstrated a good agreement between these methods of exposure assessment. In general, such studies have found that occupants tend to underestimate dampness and mold problems at home to some degree compared to inspection or dust mold measurements. This trend has been observed among both cases with disease and healthy controls. If similar under estimation applies to workplace mold exposure, non-differential misclassification of exposure would lead to some underestimation of the true effect of exposure on disease risk. As ACOS had not yet been identified at the time of the data collection for FEAS, participants were not aware of the study question on effect of dampness and mold exposures on the risk of ACOS. Mold odor is a subjective measure of dampness and mold problems, whereas experience of water damage and presence of any dampness and visible molds can be verified by other occupants. We did not specify the type of mold odor, but we asked also about the presence of odor originating from people, smoking, chemical odor and stale smell. This may have helped to identify mold odor.

Interestingly, mold odor has been the strongest exposure indicator of asthma in the published literature [9]. We conducted a systematic review and meta-analysis [9]. The summary effect estimates were 1.12 (0.98–1.27) for water damage, 1.33 (1.12–1.56) for dampness, 1.29 (1.04–1.60) for visible mold and 1.73 (1.19–2.50) for mold odor.

When investigating potential interactions between mold odor exposure and selected other exposures or personal characteristics, we found that among smokers occupational exposure to mold odor increased the risk of ACOS as high as 5.68 (95% CI 1.22–26.41). Thus, there was an indication of a synergistic effect for mold odor and smoking. Smoking is known to weaken the sense of smell [10]. This means that similar level of odor would indicate a higher exposure among smokers than non-smokers. This could also explain the higher effect estimates among smokers compared to non-smokers. When stratifying the analyses by the presence of parental atopy, we found that the significant effect of mold odor on occurrence of ACOS was restricted to those with no history of parental atopy. We did not find any explanation for this in previous literature, but we propose that those with atopic genetic propensity develop asthmatic inflammation when exposed to indoor molds, while those with no such genetic predisposition could react to such exposure, especially mold odor, with different type of inflammation leading to persistent obstruction in addition to asthma.

### Potential mechanisms

While the asthma component of ACOS is likely to be mainly related to an IgE-mediated hypersensitivity mechanism, the COPD component could be linked to non-specific inflammatory mechanisms caused by increased concentrations of mycotoxins and/or beta-d-glucan produced by molds [6, 11]. The finding that exposure to mold odor was significantly related to ACOS mainly among those with no parental history of atopy could mean that those with atopic genetic propensity develop IgE-mediated inflammation when exposed to indoor molds, which leads to rhinitis and/or asthma-only [9, 12]. In contrast, those with no genetic propensity to allergy react to mold odor exposure with different type of inflammation, which leads to persistent obstruction in addition to asthma, i.e. to ACOS. In our previous article, we compared the characteristics between ACOS and asthma-only cases [2]. Allergic diseases, especially allergic rhinitis, were less common among the ACOS cases than among the asthma-only cases. Also allergic symptoms were consistently less common among the ACOS cases than among the asthma-only cases. The ACOS cases showed or reported less often any positive allergy findings in skin prick tests or in Phadiatop analysis.

The fact that the increased risk of ACOS was detected in relation to mold odor exposure at work rather than home exposure could be explained by that people tend to repair any mold problems detected at home rather fast. In the study area altogether 64% owned their home, so they had also an economic incentive to repair any dampness problems fast. In contrast, in the workplaces the systems may be slower to react to such dampness and mold problems, so the damage may become wider and last for longer before it is repaired.

### Synthesis with previous knowledge

We have reported in 2014 from FEAS that those subjects who had newly-diagnosed asthma and were exposed to mold odor at home or at work or both had significantly reduced FEV1 level compared to those with no mold odor exposure [7]. A previous study from Scotland showed that dampness exposures at home reduce lung function significantly among adult subjects with asthma [13]. More recently, in 2015, another cross-sectional study from Scotland investigated potential relations between quantitative estimates of some biological exposures at home and Asthma Control Questionnaire, St George’s Respiratory Questionnaire as well as spirometry among 55 non-smoking adult asthmatics [14]. They reported that Environmental Relative Moldiness Index values in the homes correlated significantly with FEV1% values (correlation coefficient − 0.378, p = 0.004), while there was no correlation with concentrations of endotoxin, 1,3-beta-d-glucan or any of the dust allergens. We did not identify any previous study that had addressed the role of indoor dampness and molds as potential risk factors for ACOS.

## Conclusions

In this large population-based study of determinants of adult-onset asthma conducted in 1997–2000 in Southern Finland, we found that according to current knowledge, 6.4% of the case population had actually ACOS, which includes a component of irreversible airways obstruction. Occurrence of mold odor at work was a significant risk factor for ACOS. Thus, it is important to prevent indoor mold problems, and to repair any mold damage promptly when such problems are detected. The irreversible airways obstruction should be diagnosed and treated according to COPD guidelines, if the subject has already developed ACOS.

## Data Availability

The datasets generated and/or analysed during the current study are not publicly available due to issues of confidentiality, but are available from the corresponding author on reasonable request.

## Abbreviations

ACOS: Asthma-COPD Overlap Syndrome
COPD: chronic obstructive pulmonary disease
FEAS: Finnish Environment and Asthma Study
FEV1: forced expiratory volume in one second
FVC: force vital capacity
